# The Effect of Selected Polymorphisms of the *ACTN3*, *ACE*, *HIF1A* and *PPARA* Genes on the Immediate Supercompensation Training Effect of Elite Slovak Endurance Runners and Football Players

**DOI:** 10.3390/genes13091525

**Published:** 2022-08-25

**Authors:** Dávid Végh, Katarína Reichwalderová, Miroslava Slaninová, Miroslav Vavák

**Affiliations:** 1 Department of Track and Field, Faculty of Physical Education and Sports, Comenius University in Bratislava, Nábrežie Armádneho Generála Ludvíka, Svobodu 9, 814 69 Bratislava, Slovakia; 2 Department of Genetics, Faculty of Natural Sciences, Comenius University, Mlynská Dolina, Ilkovičova 6, 842 15 Bratislava, Slovakia

**Keywords:** supercompensation, training adaptation, sports genetics, genetic polymorphisms, sport

## Abstract

We aimed to evaluate the effect of selected polymorphisms of the *ACTN3*, *ACE*, *HIF1A* and *PPARA* genes on the immediate supercompensation training effect of elite Slovak endurance runners and football players compared with a sedentary control group. Adaptation effect levels were evaluated by 10 s continuous vertical jump test parameters measured by Optojump. Genetic polymorphisms were determined by PCR and Sanger sequencing. We found significant differences in the effect of *PPARA* genotypes in the experimental group. C allele genotypes represented an advantage in immediate supercompensation (*p* < 0.05). We observed a significant combined effect of multiple genes on immediate supercompensation (*p* < 0.05): the RR genotype of the *ACTN3* gene, the ID genotype of the *ACE* gene, the Pro/Pro genotype of *HIF1A*, and the GC and GG genotypes of *PPARA* genes. In the control group, we found a significant effect (*p* < 0.05) on immediate supercompensation of the II genotype of the *ACE* gene and the Pro/Ser genotype of the *HIF1A* gene. We found significant differences in genotype frequency of *ACE* (*p* < 0.01) and *PPARA* (*p* < 0.001) genes. We confirmed that individual genetic polymorphisms of *ACTN3*, *ACE*, *HIF1A* and *PPARA* genes have a different effect on the level of immediate supercompensation of the lower limbs depending on the training adaptation of the probands and the combination of genotypes.

## 1. Introduction

One’s level of motor ability development depends on many factors. One of the most important factors is genetics, which accounts for up to 66% of the variance in sports performance [1]. Motor abilities from the sports genetics point of view are among the traits with polygenic inheritance, which means that one phenotype results from the effect of several genes. The influence of individual genes can be different. Determining the effect of individual genes on sports performance is a difficult task, as other factors also contribute to the overall phenotype. For example, adaptation mechanisms, such as the supercompensation effect, have a significant impact, especially on motor abilities. The supercompensation effect, or supercompensation, is a complex reaction-adaptation mechanism that represents a set of responses provided by various physiological systems to an external stimulus (stress stimulus) or influence, which in our case is the external physical load. During supercompensation, there is an increase in all the levels of fitness, coordination abilities and psychological properties compared to the state before the stimulus [2]. In the professional literature, the term supercompensation is often used for the process when, after a load, depleted energy reserves are restored, and their level reaches a higher level than before the load. Supercompensation thus represents a mechanism of adaptation to stress created by an external load [3]. Supercompensation differs between individuals and also depends on the state of the adaptation itself, in which the threshold value of the stress stimulus is different for differently adapted individuals, e.g., it is different between elite athletes and beginners [4]. In terms of the speed of response and its effect, we can divide supercompensation into an immediate supercompensation effect, which occurs after just one training load, and a long-term supercompensation effect, which occurs after several training loads, or after a longer time horizon—weeks, months, even years [2,4]. During the immediate supercompensation effect, in addition to a higher supply of exhausted resources, there is also neuromuscular activation, or post-activation potentiation of the neuromuscular system, which peaks 4–15 min after a specific load [5]. Long-term supercompensation results from the cumulative effect of several training loads and thus immediate supercompensation and represents a more permanent state compared to transient physiological changes—an increased level of energy resources, hypertrophied muscles, etc. [3,6]. Genetic make-up affects and plays a significant role in the adaptability of motor abilities. Of course, genetic predisposition in itself does not automatically ensure an adapted state; it begins to adapt only after the action of an external physical load, specifically depending on the nature of the load. Different abilities and skills are needed in individual sports disciplines, in which specific gene variants can be advantageous. Athletes possessing the given variants can have higher limits and achieve better sports performances [7]. We partly understand that an individual with favourable abilities and skills for a given sports discipline, a talent for sports, has an optimal genetic makeup for a given sports performance [8]. From the point of view of elite sport, strong selection thus affects individuals also from the point of view of “sports genes” [9].

The *ACE* gene was the first gene to be linked to sports performance. The gene codes the angiotensin-1 converting enzyme, part of the RAAS system (renin-angiotensin-aldosterone system), regulating circulatory homeostasis (circulation and blood pressure), cardiovascular functions and skeletal muscle growth. The *ACE* gene has an insertion–deletion (indel) polymorphism (polymorphism code—rs4646994) with a sequence length of 287 base pairs resulting from an Alu repeat sequence that inserts into intron 16 [10,11]. This is how three *ACE* genotypes arise—II homozygote, DD homozygote and ID heterozygote. Many studies have described a higher representation of the I allele in endurance athletes, such as marathon runners, endurance athletics, rowers, mountaineers or middle- (200–400 m) and long-distance (over 400 m) swimmers, in which the frequency of the I allele increases with increasing length disciplines [12,13]. The I allele was connected with better Yo-Yo endurance test results, a higher representation of slow muscle fibres (type I), better resistance to fatigue and a higher cardiac output with better peripheral oxygenation of tissues during physical exercise [14,15,16,17,18,19]. The homozygous deletion (DD) genotype was associated with increased muscular performance in individual strength-oriented sports. It was found, for example, that the D allele was dominant in speed-strength-oriented athletes, such as sprinters and short-distance swimmers or weightlifters. The probands of the DD genotype responded to physical stress with greater hypertrophic growth of the left heart ventricle (up to 2.7 times) and greater hypertrophic and hyperplastic growth of skeletal muscles compared to the II genotype in response to exercise [20,21,22]. Williams et al. (2005) found that ACE-mediated activation of angiotensin II and deactivation of bradykinin leads to higher muscle volume, higher muscle strength and a higher proportion of type II muscle fibres [23,24].

The *ACTN3* gene codes the α-actinin 3, a structural protein of muscle fibres. This protein is specifically expressed in type II skeletal muscle fibres (mainly fast glycolytic) and is often referred to as the “speed gene” [11]. It has a connecting role between actin filaments in the Z-line region of the sarcomere, ensuring stability during contractions with a high speed and explosive character [12]. A known polymorphism of the *ACTN3* gene is the R577X (rs1815739) non-sense mutation, categorised as an SNP (single-nucleotide polymorphism), in which the C base is changed to a T, leading to a change in the amino acid sequence: arginine (R) to a premature stop codon (X). The homozygous X allele results in a complete lack of α-actinin-3 expression, which occurs in an average of 20% of the world’s population [25]. According to several studies, the X allele has a higher representation in endurance-oriented athletes, with a type I muscle fibre domination (long-distance running and swimming). The X allele also correlates with less muscle mass and lower strength. XX genotype probands have a higher VO_2max_ baseline than RR genotype carriers [17,26,27,28]. The XX genotype was also associated with increased cardiovascular fitness [29]. On the other hand, a strong correlation has been described by several studies between a higher frequency of the R allele and athletes of speed-power sports disciplines (weightlifting, sprints, short-distance swimming, etc.). In a study by Yang et al. (2003), none of the observed speed-power-oriented athletes at the Olympic Games were carriers of the XX genotype [26]. Carriers of the R allele, especially the RR genotype, had a higher representation of type II muscle fibres and showed higher muscle strength and better results in strength and speed-power [30], i.e., a better score on the seven continuous vertical jumps test, greater maximal isoinertial force, higher maximal strength and greater muscle volume after 9 weeks of lower extremity resistance training [17,28,31]. The R allele was also associated with a greater adaptive response to strength training [32].

The *PPARA* gene encodes the nuclear receptor protein PPARA (peroxisome proliferator-activated receptor α), a transcription factor [33], which is the main regulator of lipid metabolism—it supports the uptake, utilisation and catabolism of fatty acids. Furthermore, it is vital from the side of energy glucose homeostasis and vascular inflammation. It is activated in conditions of energy deprivation and metabolic and physiological stress, including physical activity in tissues that catabolise fatty acids, such as liver, skeletal (mainly type I) and cardiac muscle tissue [34]. *PPARA* has a significant role in the adaptive response to endurance training [33,35]. The most frequently analysed genetic variant is the polymorphism G/C (rs4253778). Genotypes containing the G allele were identified in endurance athletes [33], especially the GG genotype, which was associated with increased fatty acid oxidation in skeletal muscle. Its frequency was correlated with a higher proportion of slow-twitch type I muscle fibres, which use oxygen more efficiently during continuous muscle activity. Endurance athletes have relatively more slow type muscle fibres than fast type, enabling sustained muscle contraction over a more extended period [36,37]. In addition, the GG genotype has been shown to correlate with high oxygen pulse values [38]. On the other hand, the C allele was highly represented in speed-power-oriented athletes, in which the representation of type II muscle fibres was higher. Overall, C allele carriers had better anaerobic performances [39]. Athletes with the C allele, but especially with the CC genotype, had greater grip strength, showed better results in the Wingate test (WT30) and had greater muscle mass and contraction strength [31,40,41]. According to Alvarez-Romero et al. (2020), the C allele represents a significant advantage for the trainability of strength abilities [42]. The mentioned polymorphism also correlates with the growth of the left ventricle as an adaptation to external physical load, in which homozygotes of the C allele had a three-fold enlargement, and heterozygotes had a two-fold enlargement [18]. Petr et al. (2014) found statistically significant differences in maximal relative performance [Pmax·kg^−1^] in WT30 between carriers of the C allele and the GG genotype. According to their results, C allele carriers showed higher speed-strength performances in WT30 [41]. Additionally, the results show a probable metabolic advantage of the C allele in trained individuals for anaerobic metabolism [35,41].

Hypoxia is an insufficient supply of oxygen to the cells and tissues [43]. This condition is typical for strength-oriented sports disciplines, in which a lack of oxygen (exercise-induced hypoxia) is observed in the muscle tissue. The efficiency of energy supply to skeletal muscle in a hypoxic state depends on many factors affecting the “high anaerobic potential”—i.e., ATP reserves, the concentration and activity of enzymes of glycolysis and the phosphagen system, creatine phosphate, glycogen, etc. [36,44]. In human cells, oxygen homeostasis is regulated by the transcription factor—hypoxia-inducible factor-1 (HIF1) [45], which is recognised as a “master gene” that regulates the expression of downstream genes, ensuring the adaptation of cells to low oxygen partial pressure [46]. HIF1 is expressed in all human tissues, including skeletal muscle (mainly in fast glycolytic muscle fibres). HIF1 is a dimeric protein composed of the regulatory subunit HIF1A and the constitutively expressed subunit HIF1B [47]. Under normoxic conditions, HIF1A undergoes rapid degradation. Conversely, under hypoxic conditions, it is stabilised, binds to HIF1B and translocates to the nucleus, where it activates other genes in the pathway. A missense SNP polymorphism (rs11549465, C/T) of the *HIF1A* gene results in the substitution of proline at amino acid 582 to serine (Pro582Ser), and increases the stability of the HIF1A protein. This increases the hypoxic resistance of cells and improves glucose metabolism [46]. Increases in HIF1A protein (but not mRNA levels) and increased transcription of HIF1 target genes such as EPO and VEGF have been acutely demonstrated in humans during single-leg knee extensor exercise. Using hypoxia as an additional stimulus in endurance training, increased levels of *HIF1A* mRNA were found in subjects training for several weeks under conditions of high- or low-intensity muscle hypoxia. Similarly, adding training units that create hypoxic muscle conditions led to a significant modulation of the expression profile of muscle genes, which was not observed at muscle normoxia [44,48]. These data indicate that carriers of the Ser allele of the *HIF1A* gene are more predisposed to strength-oriented exercise than Pro/Pro homozygotes. Carriers of the Pro/Ser genotype in m. vastus lateralis had a significantly higher percentage of type IIX muscle fibres (fast glycolytic), mainly used in disciplines requiring a high level of explosive power. The Pro/Ser heterozygotes had a significantly higher prevalence among weightlifters and other strength-oriented athletes than in the control group [46,49,50,51].

Our selection of genes *ACE*, *ACTN3*, *HIF1A* and *PPARA* was based on the results of previous studies, as the polymorphisms’ effects on sports performance and adaptation to training load had been proven. Our gene polymorphism palette affects the composition of muscles, such as the proportion of fast and slow twitch muscle fibres, the lipid and glucose metabolism of muscles, VO_2max_ levels, oxygen consumption and adaptation to hypoxia in muscles; we presumed that these have an essential role in training adaptation in general and on immediate supercompensation. We wanted to study their effects on the immediate supercompensation individually and in combination to evaluate their significance. Moreover, as elite athletes represent adapted individuals to the training load, we wanted to assess their effects in the adapted-athlete group compared with the non-adapted control group to evaluate their role in the training adaptation process.

## 2. Materials and Methods

Subjects: The experimental group (age 23.65 ± 3.46 years; height 182.51 ± 7.25 cm; body weight 72.9 ± 7.67 kg) consisted of 64 male athletes from the highest performance class of the Slovak Republic from the following sports disciplines: endurance running (*n* = 23) and football (*n* = 41). Football players were represented by players of the 1st and 2nd Slovak football leagues. The disciplines of endurance runners included middle- (1500 m) and long-distance (3000 m, 5 km, 10 km) runs. The inclusion criteria for the experimental group were as follows: male professional or elite athlete status (national representatives, national- or international-level competitors, top athletes in the country in the sports discipline) training at least four times per week and aged 18–35. Exclusion criteria: injured or ill athletes and athletes who were not continuously training in the last six. The control group (age 19.91 ± 0.66 years, height 182.09 ± 6.03 cm, body weight 73.94 ± 10.10 kg) consisted of 54 male probands from the general population with a predominantly sedentary lifestyle within a comparable environment to the experimental group, who completed training loads as part of compulsory physical education at the university once a week. The exclusion criteria for the control group were injury, illness, and training on top of the compulsory physical education at the university. All probands were Caucasian. The recruitment of eligible participants was made continuously after the approval of the ethical committee with the help of professional football clubs, representatives and national coaches of the discipline. The recruitment of the control group was made with the help of university physical education teachers. The process and purpose of the research project were explained to the probands, who afterwards agreed to participate and signed the informed consent, which was constructed in accordance with the decision of the Ethics Committee of the Faculty of Natural Sciences, Comenius University, in Bratislava, under the registration numbers ECH19013 and ECH19015.

**Figure 1:**
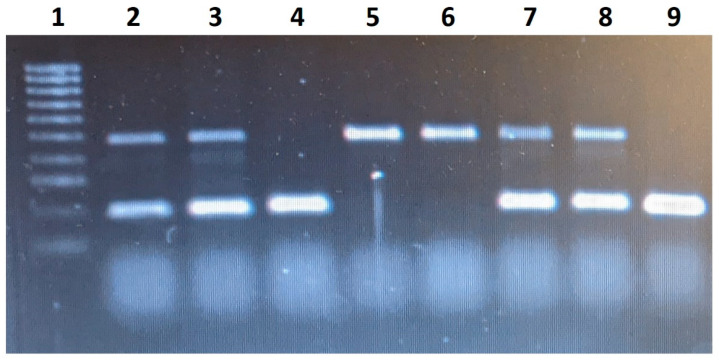
Visualization of *ACE* gene fragments after gel electrophoresis. Fragments are separated in vertical lanes; each lane represents fragments from one proband. Line 1 contains 100 bp ladder. Lanes 2, 3, 7 and 8 contain I and D fragments (visible shorter and longer bands), i.e., probands have a heterozygous ID genotype. In lanes 4 and 9, there is only a shorter fragment, i.e., probands have a homozygous genotype for allele D. In lanes 5 and 6, there are longer fragments, i.e., these probands are homozygous for the I allele.

Genotypization: DNA was isolated from the buccal swab using a standard protocol with a 5% Chelex solution. The purity and concentration of the isolated DNA were verified by spectrophotometric analysis with a nano photometer (Implen) from a sample of 1 μL of purified DNA. Based on the measured sample concentration, we determined the volume of DNA required for the subsequent PCR reaction. The volume of the PCR reaction was 20 μL, and the DNA concentration was 300–400 ng per PCR reaction. My Taq DNA polymerase (Bioline) was used with 5× My Taq Reaction Buffer (Bioline). The temperature of annellation was optimised by the gradient PCR for each gene. The PCR conditions were as follows: initial denaturation for 8 min at 95 °C, and 35 cycles with denaturation of 1 min at 95 °C, annealing for 45 s at 58 °C (*ACE*), 58.7 °C (*ACTN3* and *HIF1A*) and 50 °C (*PPARA*) with an extension for 45 s at 72 °C, and a final extension at 72 °C for 10 min. After the PCR reactions, the specificity of the products was verified on 2% agarose gel. Excluding the *ACE* gene (in which the genotypes were detected by the length of the bands on gel as stated below), the products of all other genes (*ACTN3*, *HIF1A*, *PPARA*) were then sent to a certified sequencing laboratory Microsynth Austria GmbH (ISO 9001:2015, ISO/IEC 17025:2017, STS 0429). PCR was used to detect the I and D alleles of the *ACE* gene (rs1799752) according to the method described by Tiret et al. [52] using PCR primers as follows: forward 5′-CTGGAGACCACTCCCATCCTTTCT-3′ and reverse 5′-GATGTGGCCATCACATTCGTCAGAT-3′. This method yields a PCR fragment of 190 bp and 490 bp in the presence of the D and the I alleles, respectively. Genotypes II and DD had one band of correspondent size, and genotype ID had two bands. Bands representing the different lengths of alleles of the *ACE* gene are shown in Figure 1.

**Figure 2:**
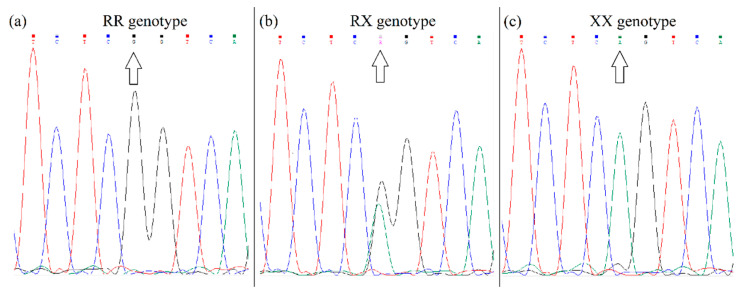
Fluorogram of *ACTN3* gene sequencing. Figure (**a**) shows the genotype RR, since nucleotide G is located at the polymorphic site. Figure (**b**) shows the genotype RX, because the nucleotides G and A are located at the polymorphic site. Figure (**c**) shows the genotype XX, since nucleotide A is located at the polymorphic site.

Genotyping of *ACTN3* R577X polymorphism (rs1815739) was performed using a PCR with primers: forward 5′-CAGCGCACGATCAGTTCAAG-3′ corresponding to a sequence in exon 15; and reverse 5′-AATCCCACGTGGAGTCTGTG-3′ corresponding to sequence in intron 15. The amplified products of 307 bp were sequenced (Microsynth). The fluorograms of the different alleles after sequencing are shown in Figure 2.

**Figure 3:**
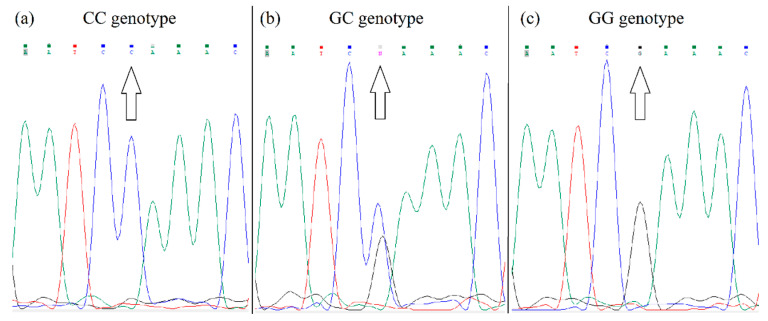
Fluorogram of *PPARA* gene sequencing. Figure (**a**) shows the CC genotype, since the polymorphic site contains the nucleotide C. Figure (**b**) shows the GC genotype, because the polymorphic site contains both G and C nucleotides. Figure (**c**) shows the GG genotype, since nucleotide G is located at the polymorphic site.

A PCR was used to detect *PPARA* gene polymorphism (rs4253778) according to the method described by Flavell et al. [53] using PCR primers as follows: forward 5′-ACAATCACTCCTTAAATATGGTGG-3′; and reverse 5′-AAGTAGGGACAGACAGGACCAGTA-3′, generating a fragment of 266 bp. The PCR products were sequenced (Microsynth). The fluorograms of the different alleles after sequencing are shown in Figure 3.

**Figure 4:**
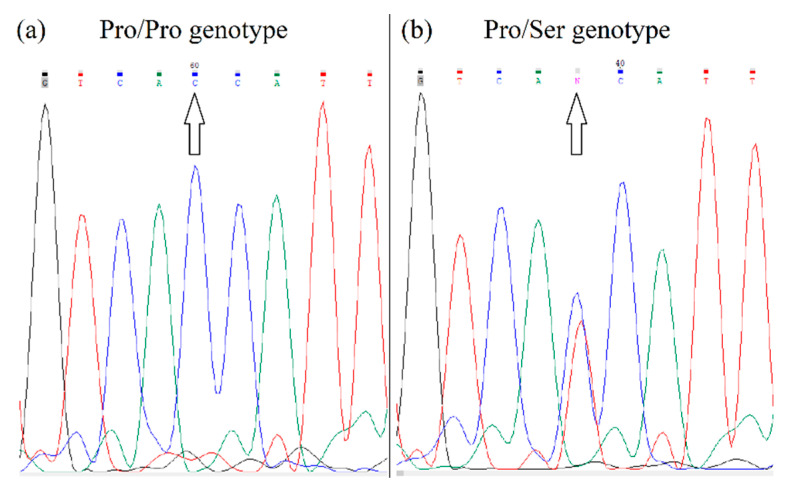
Fluorogram of *HIF1A* gene sequencing. Figure (**a**) shows the Pro/Pro genotype, since the polymorphic site contains nucleotide C. Figure (**b**) shows the Pro/Ser genotype, because the polymorphic site contains both T and C nucleotides.

Genotyping of *HIF1A* polymorphism (rs11549465) was performed using a PCR with primers: forward 5′-AGGTGTGGCCATTGTAAAAACT-3′ corresponding to sequence in intron 11; and reverse 5′-AATTCATCAGTGGTGGCAGTG-3′ corresponding to sequence in exon 12. The PCR products of 255 bp were sequenced (Microsynth). The fluorograms of the different alleles after sequencing are shown in Figure 4.

Motor tests: The immediate supercompensation effect was evaluated based on the differences in the parameters of the 10 s continuous vertical jump test (CJ10) measured by Optojump (Bolzano, Italy). The probands performed their jumps with their legs outstretched and their hands on their hips, and they tried to jump as high and take off as fast as possible [54]. By completing jumps with outstretched legs, we aimed to prevent the effect of the stretch-shortening cycle (SSC), in which the elastic abilities also play a crucial role, which we wanted to eliminate in our research due to the possibility of distorting the results [55]. They completed the tests twice, once before the training unit and once after the training unit. Before the first measurement, the participants performed a standardised warm-up used in previous research—three minutes of running at an aerobic pace, followed by two sets of ten dynamic and jumping exercises [56,57]. After the warm-up, they had a 5 min passive recovery, after which they completed the first test [56]. After the training, the probands took a second measurement after 15 min of rest. The recovery time was determined based on previous studies that observed the peak of the immediate supercompensation effect at the mentioned time after the training [3,5,6]. The training unit represented a 1–1.5 h specific load, depending on the sports discipline of the experimental group. Football players completed specific football training, while endurance runners completed specific running training. The control group had general training, which they completed as part of compulsory physical education. In the CJ10 test, we measured the average time of contact with the mat—tc [s], the average height of vertical jumps—h [cm] and the average power in the active phase of the rebound—P [W·kg^−1^]. We derived differences from the parameters of pre-training and post-training measurements—∆tc, ∆h and ∆P—based on which we evaluated the effect of immediate supercompensation.

Statistical analysis: Individual probands with different genotypes were compared with the results of the jump tests. Intragroup comparisons monitored differences based on individual genotype groups regarding ∆tc, ∆h and ∆P. To evaluate the selection pressure from the point of view of sports performance, we compared the frequency representation of individual genotypes of selected genes between the experimental and the control group. The Shapiro–Wilk test was used to verify the normality of data distribution. The chi-square (χ^2^) statistical method was used to compare the frequency of monitored genotypes between the experimental and control groups. The results from the comparison of the genotypes with the jump test parameters were statistically evaluated using the parametric statistical method ANOVA (analysis of variance) with the Bonferroni post hoc test in the computer program IBM SPSS v23 [58,59]. Similarly, comparisons of combined genotypes with jump test parameters were statistically evaluated using a multivariate analysis of variance. The level of statistical significance was set as *p* < 0.05.

## 3. Results

### 3.1. Genotype Frequency

In the experimental group, we found a statistically significant (*p* < 0.01) higher representation of both homozygous genotypes of the *ACE* gene (II and DD) at the expense of the heterozygotes compared to the control; however, in both groups of probands, the heterozygous (ID) genotype was dominating (Table 1). In the case of the *ACTN3* gene, we did not find a statistically significant difference between the genotypic distribution of individual groups of probands. In both cases, the heterozygous genotype (RX) was dominating. Still, in the experimental group, more than half of the probands were heterozygous at the expense of the XX genotype (Table 1). In the case of *PPARA*, the GG genotype was the most represented and the CC the least in both groups; however, in the case of the experimental group, we found a statistically significant (*p* < 0.001) higher proportion of the CC genotype at the expense of GG—15.63% in the experimental group and only 1.85% in the control group (Table 1). We did not find the Ser/Ser genotype of the *HIF1A* gene in either group. The distribution of the other genotypes was almost identical, with a significant dominance of the Pro/Pro genotype over the heterozygous genotype (Table 1).

### 3.2. Effect of ACTN3 Polymorphisms on the Supercompensation Effect at CJ10 in Experimental and Control Groups

We observed a reduction in differences in the experimental group, i.e., a deterioration in parameters ∆h and ∆P in post-training measurements compared to pre-training ones. In terms of ∆h, the most favourable effect was observed in the case of the RR genotype and the worst in the case of the XX genotype. In ∆P, the most favourable genotype was the RR genotype, and the worst was RX. In the control group, we observed positive values of differences, i.e., an improvement in monitored parameters ∆h and ∆P. In this case, the most favourable genotype was also the RR genotype. In ∆h, the most unfavourable was the genotype RX, while in the case of ∆P, the worst was the genotype XX (Table 2). None of the above-mentioned results were statistically significant.

### 3.3. The Effect of ACE Polymorphisms on the Supercompensation Effect in CJ10 in the Experimental and Control Groups

In the experimental group, we observed a decrease in the differences in the parameters ∆h and ∆P, except for ∆h of genotype II. In all parameters (∆h, ∆P), the II genotype was the most favourable. The most unfavourable in the case of ∆h was the ID genotype, and in the case of ∆P, the DD genotype. None of the results were statistically significant. However, if we compared the effect size using Hedges’ g statistical method, we found a statistically medium effect size in the ∆P parameters (g = 0.66) between the II and DD genotypes. In the control group, we observed positive values of differences in all monitored parameters (∆h, ∆P) for each genotype. In this case, too, the most advantageous genotype was II, and the least advantageous genotype was ID (Table 3). Of the results mentioned above, only the improvement of ∆P in the case of genotype II in the control group was statistically significant (*p* < 0.5).

### 3.4. The Effect of HIF1A Polymorphisms on the Supercompensation Effect at CJ10 in the Experimental and Control Groups

We identified only two *HIF1A* genotypes in the tested probands, namely Pro/Pro and Pro/Ser; we did not find a proband with the Ser/Ser genotype. We observed a reduction in differences in parameters ∆h and ∆P in all genotypes in the experimental group. The most favourable genotype in all parameters (∆h, ∆P) was Pro/Ser, but none of the results were statistically significant. In the case of ∆tc, we found statistically significant differences between the genotypes in the experimental group (*p* < 0.5), whereas in the case of the Pro/Ser genotype, there was a prolongation of ∆tc in the Pro/Ser comparison (by 0.013 s). In the control group, we observed positive values of differences in all monitored parameters (∆h, ∆P) for each genotype. The most favourable genotype, in this case, was Pro/Ser, in contrast to Pro/Pro in the experimental group (Table 4). Differences in ∆P between control group genotypes were statistically significant (*p* < 0.5).

### 3.5. The Effect of PPARA Polymorphisms on the Supercompensation Effect in CJ10 in the Experimental and Control Groups

We observed statistically significant differences between individual genotypes in the parameters of ∆h (*p* < 0.01) and ∆P (*p* < 0.01) in the experimental group. In the case of ∆h and ∆P, the most favourable genotype was the heterozygous genotype (GC), and the least favourable was GG. The GC and CC genotypes had positive values in the ∆h parameter compared to the GG genotype, and the differences between the GC and GG genotypes in the mentioned parameter were at the level of statistical significance *p* < 0.05. In other parameters, the probands of each genotype had already deteriorated, but as mentioned above, even in the given cases, the genotypes GC and CC had a more favourable effect in the given descending order (Table 5). We observed statistical significance at the *p* < 0.05 level when comparing ∆P parameters between the GC and GG genotypes (best and worst results). In the control group, we observed positive values of differences in all monitored parameters (∆h, ∆P) for the GG and GC genotypes, while in the case of the CC genotype, there was a decrease in the values of the ∆h and ∆P parameters. Even in the control group, the GC genotype appeared to be the most advantageous compared to other genotypes, but due to the fact that only one proband was a carrier of the CC genotype, statistical evaluation could not be completed (Table 5).

### 3.6. The Combined Effect of Genes on Selected Parameters of the Supercompensation Effect in CJ10

We statistically evaluated the individual parameters of the CJ10 test in terms of the combined effect of *ACTN3*, *ACE*, *PPARA* and *HIF1A* gene genotypes in the experimental group. We found statistically significant results in the case of parameters ∆h and ∆P. A summary of the combinations of *ACTN3*, *ACE*, *PPARA* and *HIF1A* gene genotypes and their effects, which were statistically significant, can be found in the Table 6.

## 4. Discussion

### 4.1. Genotype Frequency of ACTN3, ACE, HIF1A and PPARA Genes in Terms of Adaptation

In the case of the *PPARA* gene, we found statistically significant differences in the representation of individual genotypes, in which the representation of the CC genotype increased at the expense of the GC genotype, with an unchanged representation of heterozygotes. From the aspect of sports performance, football is generally included among disciplines with mixed sports performance [60], in which certain player positions require a higher level of speed-power abilities. In our cohort, the increased representation of the CC genotype was associated with football players, as except for one individual with the CC genotype in the group of endurance runners, all probands with the CC genotype were football players, which is in line with our expectations. Several studies described a similar higher representation of the C allele in strength- and speed-strength-oriented athletes, such as in the case of sprinters [18,39]. In contrast, the G allele was mainly represented among endurance-oriented athletes [7,33]. In the experimental group, we observed an increase in the frequency of two homozygous genotypes II and DD at the expense of heterozygotes, indicating the presence of selection pressure. Genotypes II and DD have an advantage for the two ends of the sports performance spectrum—strength (DD) and endurance (II) performance [12]. As we had endurance runners and football players in our cohort, who, depending on their playing positions, also partially fit into the group of endurance athletes, the higher representation of the II genotype matched our expectations. Other studies have also confirmed the increased representation of the I allele in endurance runners, but also in disciplines such as competing in an Ironman triathlon, cross-country skiing and swimming over medium (200–400 m) and long (over 400 m) distances [12,13]. The higher representation of the DD genotype in our probands is related to football players. We recorded 17 individuals with the DD genotype and only 9 with the II genotype out of 41 football players. The DD genotype can represent an advantage in speed-power abilities, which are essential for certain player positions with a specific sports performance. From the aspect of sports performance type, football is generally included among the disciplines with mixed sports performance [60], which partially coincides with our results, since out of the 41 football players, the mixed ID genotype occurred in 15 individuals. McAuley et al. (2021), like us, found a higher representation of the DD genotype in young football players [61]. Gineviciene et al. (2014) observed different results, as they described a higher representation of genotypes II and ID in combinations with genotypes of other genes in football players [60]. Cieszczyk et al. did not find statistically significant differences between individual genotypes when analysing the distribution of genotypes between football players and controls [46]. A higher representation of the DD genotype was described in strength- and speed-power-oriented athletes, such as sprinters, short-distance swimmers (up to 200 m) or weightlifters [12,62]. There were no statistically significant differences in genotype frequency between the groups for the *ACTN3* and *HIF1A* genes. The fact that the overall representation of genotypes did not change significantly in the case of *ACTN3* and *HIF1A* is related to the fact that their effect on the sports performance of football players and endurance runners is not so significant that the selection of genotypes would be present.

### 4.2. Immediate Supercompensation Effect in CJ10 in Terms of Gene Polymorphisms

In the case of the *ACTN3* gene, we found no statistically significant differences in terms of immediate supercompensation in either group. However, it seems that in both groups, from the side of supercompensation, the RR genotype represents an advantage for the explosive strength of the lower limbs, since in the case of the experimental group, in which performance deterioration occurred in the second measurement compared to the initial ones (negative values of the differences), the smallest deterioration was characteristic for the RR genotype group. Although the effect of the *ACTN3* gene is probably smaller compared to other genes in which we observed statistically significant differences, in the case of evaluating the effect of genotypes in combination with other genes, the RR genotype was part of every statistically significant combination and, except for one, the combinations had a positive outcome effect on the immediate supercompensation-adaptation effect (Table 6). When comparing the parameters of the experimental and control groups, the positive difference values in the control group can be evaluated as supercompensation, whereas the negative difference values in the experimental group can be evaluated as an adaptation-inhibiting effect on the deteriorating effect of physical load on sports performance. The fact that there was a supercompensatory effect in non-adapted individuals and an adaptation-inhibiting effect in the experimental group is related to the training intensity, as the experimental group had a higher training intensity. In the given case, however, it was enough for us to compare the different effects of genotypes, in which the RR genotype represented an advantage in both groups, even if the results were not statistically significant. In the case of the *ACE* gene, we found a statistically significant result only in the case of the control group, namely in the case of the II genotype (Table 3). The II genotype represented an advantage in both groups in terms of the supercompensation effect. In the control group, supercompensation occurred in each group, whereas in the experimental group, partial supercompensation (positive ∆h, negative ∆P) occurred only in the case of the II genotype. After evaluating the effect size using Hedges’ g in the case of the experimental group, we found a statistically significant result—a medium effect size when comparing the II with the DD genotype. Our results supported the results of other studies, according to which II individuals had better results in the Yo-Yo endurance test, higher peripheral tissue oxygenation during physical exercise and generally better fatigue resistance compared to the control [14,17,18,19]. A higher level of endurance abilities is probably also related to the maintenance of higher speed-power performance even after the training load. We found statistically significant differences in the *HIF1A* gene in the control group, in which the Pro/Ser genotype showed better parameters in terms of an immediate supercompensation effect on the anaerobic lactate performance of the lower limbs (Table 4). We did not find statistically significant differences in the experimental group, even though in ∆P, it seemed that the Pro/Pro genotype represented an advantage in the given case. The fact that the opposite effect of genotypes was observed between the adapted and non-adapted group points to the important influence of the long-term supercompensation effect in the case of the *HIF1A* gene. Our results indirectly confirm studies that observed a higher frequency of the Pro/Pro genotype in endurance athletes [12,51]. In the experimental group, we found the most statistically significant results in immediate supercompensation in the case of the *PPARA* gene in the experimental group. Groups with the C allele, i.e., the GC and CC genotypes, between which there were no statistically significant differences in the measured parameters, had an advantage over the GG genotype group (Table 5). C allele groups achieved supercompensation in the ∆h parameter (positive values of the differences), while in the case of the GG genotype, there was a significant deterioration in the differences. Although at first glance, it may seem that the CC genotype has the greatest deterioration in the observed parameters in the control group, the given group is represented by a single proband. Therefore, it was not possible to statistically evaluate the result. However, if we look at the GC and CC genotypes, in the case of the GC genotype, the probands showed a higher supercompensation compared to the GC, which correlates with the results from the experimental group (Table 5). Our results are supported by the result of Alvarez-Romero et al. (2020), which describes a better trainability (adaptation) of strength abilities in the case of the C allele [42]. Other studies found a correlation between C allele carriers and a higher level of anaerobic performance, better parameters of the WT30 test and greater muscle mass and strength, which support our results [39,40,41].

Since sports performance represents polygenic inheritance from a genetic point of view, in which the effect of genotypes of different genes is combined, we also analysed the effect of polymorphisms in combinations. We observed statistically significant results of the combined effect on the parameters of the CJ10 test, in which individual combinations were composed of genotypes R/(*ACTN3*), I/(*ACE*), Pro/Pro (*HIF1A*) and C/(*PPARA*). In the case of the combination of RR (*ACTN3*) + ID (*ACE*) + GG (*PPARA*) genotypes, there was a negative effect on supercompensation. The fact that, in the given case, two advantageous genotypes were combined with one disadvantageous one and the resulting effect on the observed trait was negative, is probably related to the fact that many combinations did not occur in our cohort. Additionally, the fact that some allele combinations did not appear in the experimental group indicates a possible genetic selection force in elite sports. Similar research, in which the effect of combined genotypes on adaptation to strength load was compared, was conducted by Ahmetov et al. [31]. The advantageous combination of genotypes in adaptation was R/(*ACTN3*) + D/(*ACE*) + C/(*PPARA*), which coincides with our results; in our case, it was the combination of genotypes RR (*ACTN3*) + ID (*ACE*) and CG (*PPARA*).

Our study had some limitations, including the size of the cohorts. In small groups, it happens that a rare genotype occurs only in a few individuals or does not occur at all. It can happen even more often when the genotypes of multiple genes are combined. The results could be better interpreted if more individuals were in each tested group. However, the selection force can play a significant role by eliminating the individuals with non-favourable alleles. In addition, different sports disciplines and even different player positions in football may require different motor abilities, even if the type of the sports performance is comparable, which can also lead to a distortion of the result. As sports performance is a polygenic trait, more gene polymorphisms should be included in future works simultaneously to evaluate their combined effect.

## 5. Conclusions

Our results show that the individual effects of *ACE*, *HIF1A* and *PPARA* gene polymorphisms are primarily influenced by adaptation or long-term supercompensation. In the non-adapted (control) group, genotype II (*ACE*) and Pro/Ser (*HIF1A*) had an advantage for immediate supercompensation, while in the adapted (experimental), the C/ genotype (*PPARA*) had the advantage. Based on the effect size, in the adapted group, the advantageous genotype from the side of supercompensation was the II genotype (*ACE*). The fact that the individual genotypes of the *PPARA* and *ACE* genes can have a significant effect on sports performance, even individually, could be seen in the frequency analysis of the individual genotypes, in which there were statistically significant differences in representation between the experimental and control groups. It can be an example of the genetic selection force, in which the advantageous genotype frequency is increased: CC (*PPARA*), as well as II and DD (*ACE*). However, our most important result is that in adapted individuals, in addition to the genotypes whose effect also appears individually (*ACE* and *PPARA*), the combination of the *ACTN3* genes together with *HIF1A* has an essential role in the resulting phenotype. For this reason, it is necessary to evaluate the combined effect of genes on the genetic predisposition for a particular trait. In practice, these results can be used in sports performance genetic predisposition modelling. From the point of view of the coaches and teams, it could be used to identify elite athletes with high potential. Still, from the point of view of the individuals, it could be used to identify the appropriate sports discipline and adjust the training load and the time needed for regeneration, improving performance and preventing injuries.

## Figures and Tables

**Table 1:** Frequency of genotypes and their comparison between experimental and control groups.

Gene	Genotype	Experimental Group (*n* = 64)	Control Group (*n* = 54)	χ^2^	Sig. Level
		Quantity (Frequency)	Quantity (Frequency)		
*ACE*	II	15 (23.44%)	9 (16.67%)	9.266	*p* < 0.01
	ID	26 (40.63%)	32 (59.26%)		
	DD	23 (35.94%)	13 (24.07%)		
*ACTN3*	RR	24 (37.50%)	20 (37.04%)	3.979	*p* = 0.11
	RX	34 (53.13%)	24 (44.44%)		
	XX	6 (9.38%)	10 (18.52%)		
*PPARA*	GG	37 (57.81%)	39 (72.22%)	67.7	*p* < 0.001
	GC	17 (26.56%)	14 (25.93%)		
	CC	10 (15.63%)	1 (1.85%)		
*HIF1A*	Pro/Pro	53 (82.81%)	47 (87.04%)	1.018	*p* = 0.25
	Pro/Ser	11 (17.19%)	7 (12.96%)		

Legend: χ^2^—chi-square, sig.—significance. The table shows the frequency of individual genotypes of selected genes in the experimental and control groups. We compared the representation of the genotypes between the experimental and control groups by the chi-square test (χ^2^), and the results are shown in the table with their significance level.

**Table 2:** Effect of the *ACTN3* gene on the supercompensation effect in CJ10.

	Experimental Group (*n* = 64)			Control Group (*n* = 54)		
Alleles	RR	RX	XX	RR	RX	XX
*n* (%)	24 (37.5)	34 (51.13)	6 (9.38)	20 (37.04)	24 (44.44)	10 (18.52)
∆t_c_ [s]	0.012 (0.023)	0.010 (0.018)	−0.002 (0.011)	0.004 (0.015)	0.000 (0.015)	0.001 (0.018)
∆h [cm]	−0.09 (3.17)	−0.86 (2.51)	−1.38 (2.40)	1.30 (3.54)	0.60 (2.91)	1.00 (3.97)
∆P [W·kg^−1^]	−1.55 (4.15)	−2.25 (3.86)	−1.70 (3.82)	0.98 (2.87)	0.76 (2.63)	0.72 (3.23)

The table shows the frequency of the *ACTN3* gene alleles in the experimental and control groups (*n*—number). The effect of immediate supercompensation was evaluated based on the differences derived from the CJ10 test parameters of pre-training and post-training measurements—average contact time differences (∆t_c_), average jump height differences (∆h) and average power differences (∆P). Values in parentheses at ∆t_c_, ∆h and ∆P represent standard deviation. The results from the intragroup comparison of genotypes with jump test parameters were statistically evaluated using the parametric statistical method ANOVA (analysis of variance) with the Bonferroni post hoc test.

**Table 3:** Effect of *ACE* gene on supercompensation effect in CJ10.

	Experimental Group (*n* = 64)			Control Group (*n* = 54)		
Alleles	II	ID	DD	II	ID	DD
*n* (%)	15 (23.44)	26 (40.63)	23 (35.94)	9 (16.67)	32 (59.26)	13 (24.07)
∆t_c_ [s]	0.005 (0.018)	0.004 (0.017)	0.017 (0.022)	0.003 (0.016)	0.003 (0.015)	0.001 (0.016)
∆h [cm]	0.08 (3.38)	−0.99 (2.71)	−0.66 (2.40)	2.32 (1.80)	0.23 (3.58)	1.72 (3.11)
∆P [W·kg^−1^]	−0.32 (4.53)	−1.85 (3.26)	−3.09 (3.96)	2.30 (2.25) *	0.06 (2.77)	1.73 (2.62)

The table shows the frequency of the *ACE* gene alleles in the experimental and control groups (*n*—number). The effect of immediate supercompensation was evaluated based on the differences derived from the CJ10 test parameters of pre-training and post-training measurements—average contact time differences (∆tc), average jump height differences (∆h) and average power differences (∆P). Values in parentheses at ∆t_c_, ∆h and ∆P represent standard deviation. The results from the intragroup comparison of genotypes with jump test parameters were statistically evaluated using the parametric statistical method ANOVA (analysis of variance) with the Bonferroni post hoc test. * represents *p* < 0.05.

**Table 4:** Effect of *HIF1A* gene on supercompensation effect in CJ10.

	Experimental Group (*n* = 64)		Control Group (*n* = 54)	
Alleles	Pro/Pro	Pro/Ser	Pro/Pro	Pro/Ser
*n* (%)	53 (82.81)	11 (17.19)	47 (87.04)	7 (12.96)
∆t_c_ [s]	0.007 (0.020) *	0.020 (0.013) *	0.001 (0.015)	0.007 (0.017)
∆h [cm]	−0.62 (2.71)	−0.62 (3.16)	0.69 (3.20)	2.59 (3.83)
∆P [W·kg^−1^]	−1.71 (3.91)	−3.02 (3.96)	0.50 (2.58)	3.08 (3.29) *

The table shows the frequency of the *HIF1A* gene alleles in the experimental and control groups (*n*—number). The effect of immediate supercompensation was evaluated based on the differences derived from the CJ10 test parameters of pre-training and post-training measurements—average contact time differences (∆t_c_), average jump height differences (∆h) and average power differences (∆P). Values in parentheses at ∆t_c_, ∆h and ∆P represent standard deviation. The results from the intragroup comparison of genotypes with jump test parameters were statistically evaluated using the parametric statistical method ANOVA (analysis of variance) with the Bonferroni post hoc test. * represents *p* < 0.05.

**Table 5:** Effect of *PPARA* gene on supercompensation effect in CJ10.

	Experimental Group (*n* = 64)			Control Group (*n* = 54)		
Alleles	GG	GC	CC	GG	GC	CC
*n* (%)	37 (57.81)	17 (26.56)	10 (15.63)	39 (72.22)	14 (25.93)	1 (1.85)
∆t_c_ [s]	0.011 (0.017)	0.003 (0.021)	0.012 (0.026)	0.001 (0.017)	0.004 (0.011)	0.017
∆h [cm]	−1.13 (2.80) *	0.09 (2.47) *	0.06 (2.94)	0.76 (3.59)	1.66 (2.34)	−2.41
∆P [W·kg^−1^]	−2.91 (3.99) *	−0.16 (2.58) *	−1.36 (4.66)	0.79 (2.87)	1.24 (2.44)	−3.34

The table shows the frequency of the *PPARA* gene alleles in the experimental and control groups (*n*—number). The effect of immediate supercompensation was evaluated based on the differences derived from the CJ10 test parameters of pre-training and post-training measurements—average contact time differences (∆t_c_), average jump height differences (∆h) and average power differences (∆P). Values in parentheses at ∆t_c_, ∆h and ∆P represent standard deviation. The results from the intragroup comparison of genotypes with jump test parameters were statistically evaluated using the parametric statistical method ANOVA (analysis of variance) with the Bonferroni post hoc test. * represents *p* < 0.05.

**Table 6:** Combined effect of *ACTN3*, *ACE*, *PPARA* and *HIF1A* gene genotypes on selected parameters of the supercompensation effect at CJ10 in the experimental group.

Parameter	Gene (Genotype)					Effect	Significance Level
∆h	*ACTN3* (RR)	+	*ACE* (ID)			Positive	*p* < 0.05
	*ACTN3* (RR)	+	*PPARA* (GC)			Positive	*p* < 0.05
	*ACTN3* (RR)	+	*ACE* (ID)	+	*HIF1A* (Pro/Pro)	Positive	*p* < 0.05
	*ACTN3* (RR)	+	*ACE* (ID)	+	*PPARA* (GG)	Negative	*p* < 0.05
∆P	*ACTN3* (RR)	+	*ACE* (ID)	+	*HIF1A* (Pro/Pro)	Positive	*p* < 0.05

The table shows the combinations of genotypes with a statistically significant effect on parameters of average jump height differences (∆h) and average power differences (∆P) in the experimental group. Comparisons of combined genotypes with jump test parameters were statistically evaluated using a multivariate analysis of variance.
